# Improving Critical Appraisal Teaching Experience for Psychiatry Resident Doctors: A Multi-Cycle Quality Improvement Project

**DOI:** 10.1192/bjo.2026.11485

**Published:** 2026-06-30

**Authors:** Hardeep Singh, Mary-Anne Cotton, Sidra Shaheed, Asuvathan Aravinthan, Aisha Anwar

**Affiliations:** 1 Dorset Healthcare University NHS Foundation Trust, Poole, United Kingdom; 2 Surrey and Borders NHS Foundation Trust, Surrey, United Kingdom

## Abstract

**Aims:**

To evaluate and improve the Critical appraisal teaching experience for residents at Surrey and Borders NHS Foundation Trust (Foundation year, GP and Core Psychiatry Trainees) within the SABP East Locality using a structured, iterative quality improvement approach, and to enhance trainee confidence in preparation for MRCPsych examinations and clinical application of the critical appraisal skills.

**Methods:**

A multi-cycle project was carried out in line with the quality improvement framework between October 2024 and July 2025. A baseline survey within the SABP East locality assessed the confidence of trainees in critically appraising research and their views on critical appraisal teaching. Pre- and post-cycle surveys were conducted, and changes in trainee confidence were measured across multiple critical appraisal domains. Based on survey insights, interventions were introduced across three cycles, including restructuring journal club sessions through embedding peer-led critical appraisal teaching, systematically using critical appraisal skill programme checklists, and developing supporting online learning resources. This model was adapted and further rolled out to other SABP localities following successful implementation within the SABP East locality.

**Results:**

There was a progressive improvement in self-reported confidence across all cycles. Overall confidence in critically appraising research increased from approximately 50–55% at baseline to nearly 80% following cycle III. Improvements were seen across appraisal of diagnostic, prognostic, qualitative, and randomised control trial designs, as well as systematic reviews. The structured journal club format, use of Critical Appraisal Skill Program checklists, and accessible online resources were consistently rated positively by trainees.

**Conclusion:**

Restructuring of the Journal club sessions with a focus on the peer-led critical appraisal teaching model improved trainee confidence and engagement with evidence-based medicine. Incorporating the Critical Appraisal Skill Program checklists provided a consistent, practical appraisal framework, with online resources supporting independent learning. Successful implementation within the SABP East locality further enabled the rollout of this approach across other SABP localities. Future work will aim to evaluate implementation and impact across all localities, including longer-term effects on training outcomes, success in MRCPsych examinations and clinical decision-making. However, findings should be interpreted in the context of trainee turnover, as only core psychiatry trainees contributed consistent longitudinal data.

